# GM-CSF Promotes Immune Response and Survival in a Mouse Model of COVID-19

**DOI:** 10.21203/rs.3.rs-1213395/v1

**Published:** 2022-01-26

**Authors:** L.V. Kendall, T.D. Boyd, S.H. Sillau, A. Bosco-Lauth, N. Markham, D. Fong, P. Clarke, K.L. Tyler, H. Potter

**Affiliations:** 1. Colorado State University, Department of Microbiology, Immunology and Pathology, Fort Collins, CO; 2. University of Colorado Alzheimer’s and Cognition Center, Aurora, CO; 3. Linda Crnic Institute for Down Syndrome, University of Colorado School of Medicine, Aurora, CO; 4. Department of Neurology, University of Colorado School of Medicine, Aurora, CO; 5. Colorado State University, Department of Biomedical Sciences, Fort Collins, CO; 6. Department of Pathology, University of Colorado Anschutz School of Medicine, Aurora, CO; 7. Denver VA Medical Center, Denver CO; 8. Departments of Immunology and Microbiology, and Medicine, University of Colorado School of Medicine, Aurora, CO

## Abstract

COVID-19 results in increased expression of inflammatory cytokines, but inflammation-targeting clinical trials have yielded poor to mixed results. Our studies of other disorders with an inflammatory component, including Alzheimer’s disease, chemobrain, Down syndrome, normal aging, and West Nile Virus infection, showed that treatment with the ‘pro-inflammatory’ cytokine granulocyte-macrophage colony stimulating factor (GM-CSF) in humans or mouse models alleviated clinical, behavioral, and pathological features. We proposed that human recombinant GM-CSF (sargramostim) be repurposed to promote both the innate and adaptive immune responses in COVID-19 to reduce viral load and mortality^1^. Here, we report the results of a placebo-controlled study of GM-CSF in human ACE2 transgenic mice inoculated intranasally with SARS-CoV2 virus, a model of COVID-19. Infection resulted in high viral titers in lungs and brains and over 85% mortality. GM-CSF treatment beginning one day after infection increased anti-viral antibody titers, lowered mean lung viral titers proportionately (p=0.0020) and increased the odds of long-term survival by up to 5.8-fold (p=0.0358), compared to placebo. These findings suggest that, as an activator of both the innate and adaptive immune systems, GM-CSF/sargramostim may be an effective COVID-19 therapy with the potential to protect from re-infection more effectively than treatment with antiviral drugs or monoclonal antibodies.

There is much debate about how to treat disorders that include an inflammatory component. In COVID-19, for example, it is unclear whether inflammation plays a deleterious role and should be targeted, or whether instead it should be employed as part of a strategy of recruiting the immune system against the virus, as we proposed^1^. Previously, we investigated this approach to treating multiple disorders by stimulating the innate immune system to aid the body’s healing and regenerative mechanism(s). Specifically, in Alzheimer’s disease (AD), inflammation is evident by the activation of microglia and astrocytes, the consequent over-expression of inflammatory cytokines and acute phase proteins in both the brain and blood^2–4^. These observations led to the inference that inflammation is an important contributor to AD and were reinforced by the finding that people with rheumatoid arthritis (RA) have a much lower risk of developing AD, which was attributed to their use of non-steroidal anti-inflammatory drugs (NSAIDs), which thus might be employed to treat AD^5^. However, NSAIDs were unsuccessful and potentially detrimental in treatment trials of AD and mild cognitive impairment participants^5–7^.

Hypothesizing instead that RA might induce a natural resistance to AD, independent of NSAID use, we searched for blood-borne molecules in RA patients that might explain this protective effect, focusing on cytokines that activate the innate immune system, which might, for example, activate phagocytes to remove amyloid deposits from the brain. By interrogating many molecules of potential interest by logic and experimentation, we found that treatment of mouse models of AD with GM-CSF, a cytokine upregulated in RA blood, resulted in activation of microglia, removal of amyloid, and restoration of learning and memory to normal^8^, which findings were later replicated by others^9^. GM-CSF is traditionally considered to be pro-inflammatory, but is also an innate immune system stimulant and an activator of antigen presentation, and recombinant human GM-CSF (rhGM-CSF/sargramostim) has been FDA approved for 30 years to stimulate hematopoiesis to treat leukopenia^1^.

Recently, we showed in a phase II clinical trial of mild-moderate AD participants that three weeks of sargramostim treatment improved cognition (measured by the Mini Mental State Exam) and partially normalized the levels of AD-related blood biomarkers of neuropathology and neurodegeneration (Aβ-40, Total Tau, UCH-L1) compared to baseline and to placebo^10^. We have also found that GM-CSF treatment leads to: 1) improved cognition and decreased neuropathology in a mouse model of Down syndrome^11^, 2) improved cognition in aged wild-type mice^8,11^, since replicated by others^12^, and is associated with 3) improved cognition in leukemia patients with chemotherapy-associated cognitive impairment after immune system chemoablation and hematopoietic cell transplantation^13^. Furthermore, Parkinson’s disease mouse models and participants in a small phase I trial benefitted from GM-CFS/sargramostim treatment^14^. Although these disorders and models all exhibit markers of inflammation, none develop AD pathology, suggesting that GM-CSF has broad immune and cognition-enhancing and/or neuron-protective activity^1,15^.

Many viral diseases, including West Nile Virus (WNV) encephalitis, bacterial and viral pneumonia, Severe Acute Respiratory Syndrome (caused by SARS-CoV and Sars-CoV-2) and Middle East Respiratory Syndrome, exhibit markers of inflammation, including in the brain, from which it has been inferred that an effective treatment should include inhibiting inflammation, for example using steroids^16^. Although dexamethasone has been used in patients with severe SARS-CoV-2-caused COVID-19 to reduce hyper-inflammation, recent evidence contradicts the presence of such a ‘cytokine storm’ because inflammatory cytokines are no more expressed during COVID-19 than during other respiratory diseases or sepsis^17–20^, and clinical trials seeking to block IL-6 signaling, the favored target in the COVID-19 ‘cytokine storm’, have disappointed^21^.

Instead, we suggested that viral diseases, including WNV encephalitis and COVID-19 may be more effectively treated by *increasing* the activation of the innate immune system with injected and/or inhaled GM-CSF/sargramostim^1^. This use of GM-CSF was partly supported by the previous finding that in WNV- and Japanese Encephalitis virus-infected mice, *reducing* microglia and macrophages by blocking the receptor for another cytokine, macrophage colony-stimulating factor, with PLX5622 resulted in increased viral load and mortality^16^, suggesting that, rather than reflecting or inducing a dangerous pro-inflammatory milieu, macrophages and microglia might instead be beneficial in fighting the viral infection. In preliminary experiments in mice infected by footpad injection with the TX02 strain of WNV, we found that daily GM-CSF treatment starting one day after viral inoculation improved survival to 80% from approximately 35% in placebo-treated mice (p=0.0034) (^1^; Stonedahl, Clarke, Potter, Boyd, Tyler, in preparation). Subsequently, and possibly independently of our proposal, several clinical trials were initiated to test the potential of GM-CSF/sargramostim as a treatment for COVID-19 (NCT04326920, NCT04400929, NCT04411680, NCT04569877), although several proposed to block GM-CSF to combat the cytokine storm, but found no or little benefit (see NCT04351152).

To test the potential of GM-CSF to treat COVID-19, we utilized transgenic mice that express the human *ACE2* gene under the control of the keratin-18 promoter (B6.Cg-Tg(K18-ACE2)2Prlmn/J), which is required for coronaviruses to enter and infect cells^22–26^.

## Weight Loss in SARS-CoV-2 Infected Mice

Based on published morbidity, weight loss, and mortality results of K18-hACE2 mice infected intranasally with SARS-CoV-2 virus^22–27^, we chose an inoculum of 10^4^ PFU to assure that some mice would survive until the end of the experiment on day 14 post-infection. GM-CSF (200 mcg/kg in 200 μL) or saline treatment was begun one day post-infection and continued daily through day 14, when surviving mice were sacrificed. We carried out two consecutive experiments with the only difference being that the 10^4^ PFU inoculum was instilled into the nose in a 50 μL volume for cohort 1 and changed to a 25 μL volume for cohort 2 to make instillation easier. As discussed below, this change resulted in substantially less viral infection.

Supplemental Tables S1 and S2 show the weights and mortality of each mouse in cohorts 1 and 2, respectively, at each day post-infection (DPI). Viral titers (plaque forming units, PFU) were measured per 100 μg of lung or brain tissue at death or euthanasia due to morbidity, or at sacrifice if the mouse survived until day 14. Two GM-CSF-treated mice (one each in cohort 1 (#1-15) and cohort 2 (#2-21)) died immediately after the treatment injection, and were censored in the subsequent analyses of virus-dependent mortality because their deaths could not be attributed to the infection.

**Figure 1.**
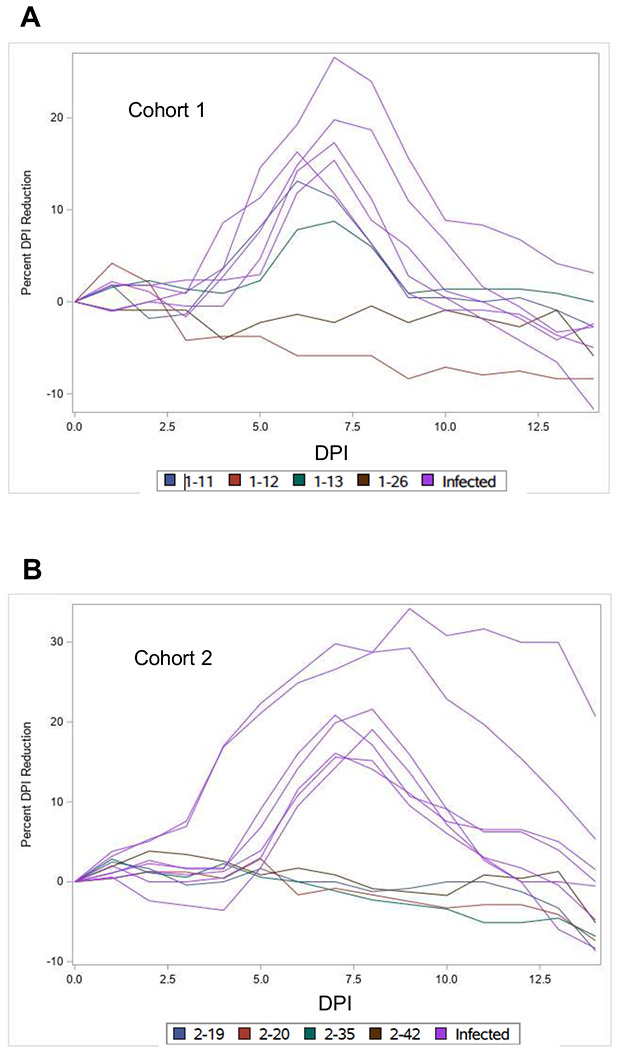

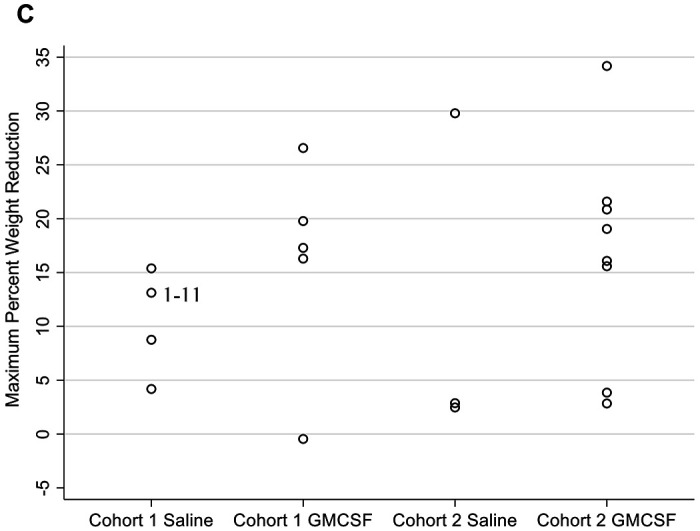
Weight Loss Reflects Effective SARS-CoV-2 Infection in K18-hACE2 Mice. Spaghetti plots of % weight loss at each day post infection (DPI)_of mice in cohort 1 (**A**) and cohort 2 (**B**) that survived until the end of the experiment on day 14. In cohort1, it is evident that mice #1-12 (saline) and #1-26 (GM-CSF) did not lose any weight and were not infected effectively at all, and that mouse #1-13 (saline) showed minor weight loss and was very poorly infected. Mouse #1-11 (saline) was borderline poorly infected (13.1% maximum weight loss followed by rapid improvement. For analyses of mortality (Figure 3), data from mice #1-12, #1-13, #1-26, were not included, and calculations were made in which mouse #1-11 was considered either infected and its data included or not infected, and its data not included. In cohort 2, it is evident that mice #2-19 (saline), and #2-20 (saline) #2-35 (GM-CSF), #2-42 (GM-CSF) did not get infected effectively. Poor infection was confirmed by low levels of antiviral antibodies (Figure 2D). Of the seven mice in cohort 2 that survived to day 14 and showed evidence of effective infection due to weight loss, six had been treated with GM-CSF, and one had been treated with saline. **C**. Dot plots showing the maximum % weight loss of each mouse that survived until the end of the experiment on day 14 in cohort 1 and cohort 2, either treated with GM-CSF or saline, as indicated.

Figures 1A and 1B show spaghetti plots of the % weight loss over time compared to baseline for each mouse in cohort 1 and 2, respectively that survived through the end of the experiment on day 14. Figure 1C shows dot plots of the maximum % weight loss of the surviving mice in cohorts 1 and 2 for each treatment. Based on them not losing any weight (ie. being asymptomatic), mice #1-12 (saline), #1-26 (GM-CSF), #2-19 (saline), and #2-20 (saline) #2-35 (GM-CSF), #2-42 (GM-CSF), evidently did not get infected effectively, and mouse #1-13 (saline), which showed minor weight loss, was very poorly infected. Data from these mice were not included in our subsequent analyses. Based on relatively minor weight loss in Figures 1A,C and Table S1, mouse #1-11 (saline) was borderline poorly infected (13.12% maximum weight loss). For mortality analyses, separate calculations were made in which mouse #1-11 was included or not included. All other surviving mice exhibited 15.58%-34.18% maximum weight loss.

## GM-CSF Reduces Lung Viral Titer

Tables S1 and S2 show the viral titers in lung and brain tissues of mice from cohorts 1 and 2 that died or were euthanized during the course of the experiment or that survived and were sacrificed on day 14. All mice that survived until day 14 had completley cleared the virus from both their lungs and brains (<10 PFU/100 μg tissue), whether they received GM-CSF or saline treatment or whether they were in cohort 1 or 2. One striking result is that the mean lung viral titers of the non-surviving saline-treated control mice from cohorts 1 and 2 were 2.05x10^5^ and 2.09x10^4^, respectively, whereas the mean brain viral titers were 2.3x10^14^ and 3.3x10^7^, respectively. Thus, a much higher viral titer developed in brain tissue compared to lung tissue in the control mice in each cohort, and orders of magnitude higher viral titers developed in both brain tissue and lung tissue in the control mice in cohort 1 vs. cohort 2. We surmised that the differences in viral titers between the two cohorts arose from the 25 μL instillation volume used for cohort 2 being too small to effectively pass the nasal cavity to reach the lung. A review of the literature supported this interpretation^28,29^. This seemingly minor difference in the inoculation method not only led to differences in the virus titers but also affected the mortality results below.

**Figure 2.**
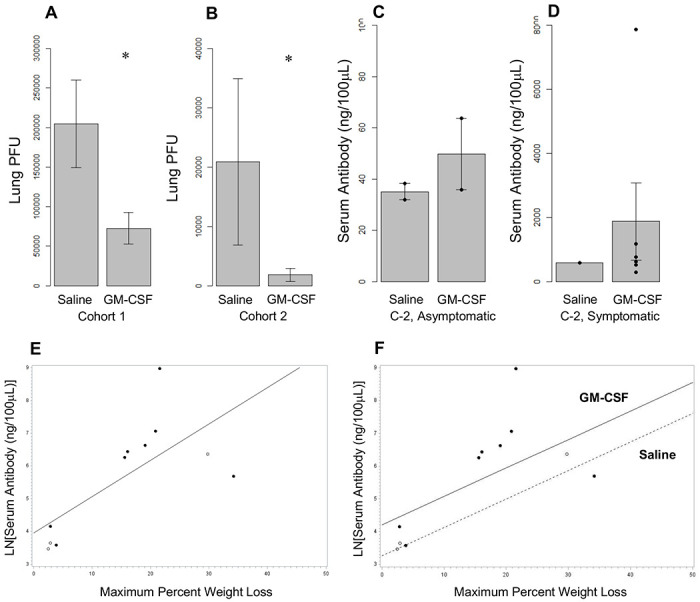
GM-CSF Treatment Lowers Viral Titers in Lung Tissue in Non-Surviving SARS-CoV-2-Infected K18-hACE2 Mice and increases Anti-Viral Antibody Titers in the Sera of the Surviving mice. As discussed in the text, all surviving mice (GM-CSF and saline-treated) had completely cleared the SARS-CoV-2 virus. Plots (mean/SEM) are shown of the viral titers in lung tissue (PFU/100 μg of tissue) from the GM-CSF- and saline-treated non-surviving mice in cohort 1 (**A**) and cohort 2 (**B**). The lung viral titers in cohort 1 were an order of magnitude higher than those in cohort 2 which received the 10^4^ PFU inoculum instilled into the nose in half the volume as was used for cohort 1, as discussed. Because of the large difference in mean viral titers in the two cohorts, the titer for each GM-CSF-treated mouse was statistically compared to the mean titer of the saline-treated mice in each cohort, which also allowed an overall treatment effect to be determined. GM-CSF treatment reduced lung viral titers, proportionately, in cohort 1 (p value = 0.0156) and cohort 2 (p value = 0.0108) and in both cohorts combined (p value = 0.0020). Brain viral titers were not different between GM-CSF and saline (cohort 1: p=0.5212; cohort 2: p=0.9083; not shown). (**C,D**) Anti-viral antibody levels in the sera obtained from many of the mice in cohort 2 were determined by ELISA (KRISHGEN Biosystems). With a few exceptions, all mice that died during the course of the experiment had not made detectable antibodies at death (Table S2). Mice that were asymptomatic and thus deemed poorly infected based on their not losing any weight, did develop some anti-viral antibodies, especially if treated with GM-CSF, indicating that they had been exposed to the virus but did not get effectively infected (**C**;mean/SEM). Mice that lost substantial weight and recovered developed more antibodies (**D**). The average antibody level of the 6 GM-CSF treated, well-infected, surviving mice was over three times that of the single saline-treated mouse that was well infected and survived to 14 days. (**E**) Correlation analysis of maximum % weight loss and LN antibody titers of all surviving mice, both well and poorly infected and treated with GM-CSF (filled circles) or saline (open circles) (Table S2), indicates that mice responded to increased weight loss morbidity due to viral infection with increased antibody production (Pearson correlation coefficient (log scale for serum antibody) = 0.71 (p value = 0.0145)). (**F**) Separate regression lines were fit by treatment. A common residual variance across treatment groups was imposed because of the small sample and lack of scatter for the saline group. The estimated saline slope was 1.0345 units on the natural logarithm of serum antibody, or a change of 181.356% in the serum antibody, per 10 percent units of maximum weight loss (p value = 0.1340). The estimated GM-CSF slope was 1.0006 units on the natural logarithm of serum antibody, or a change of 171.992% in the serum antibody, per 10 percent units of maximum weight loss (p value = 0.0890). As the resulting slopes estimates were similar there was no evidence a difference (p value = 0.9672), the model was simplified to an additive one, with a common slope by treatment group, parallel lines. The estimated common slope was 1.0144 units on the natural logarithm of serum antibody, or a change of 175.776% in the serum antibody, per 10 percent units of maximum weight loss (p value = 0.0239). Predicted serum antibody was 1.0969 units greater in GM-CSF compared to saline on the natural logarithmic scale (a ratio of serum antibody of 2.9949) (p value = 0.2458). Statistically power and reliability are limited by the small sample size, especially for the saline group.

An anti-viral effect of GM-CSF is also indicated in Tables S1 and S2 and in Figure 2A,B. The mean lung viral titers of the non-surviving GM-CSF-treated mice of cohorts 1 and 2 were 7.3x10^4^ and 1.8x10^3^, respectively, substantially lower than in the lungs of the corresponding saline-treated mice, proportionately. Compared to saline treatment, GM-CSF treatment resulted in reduced viral titers in the lung in cohort 1 (64.58%, (p=0.0156) and cohort 2 (91.32%, p=0.0108) and also when the data from the two cohorts were combined (84.77%, p=0.0020). Viral titers in the brain showed no GM-CSF treatment effect.

## GM-CSF Increases Anti-Viral Antibodies

Anti-SARS-CoV-2 spike antibodies in the sera of mice of cohort 2 were measured at death/euthanasia/sacrifice (Table S2). Mice that died during the experiment developed few to no detectable antibodies at death. Mice that survived to sacrifice at day 14, but were judged to be poorly infected due to lack of weight loss, generated few antibodies, validating weight loss as a surrogate marker of successful infection. The asymptomatic surviving mice that received GM-CSF developing an estimated 1.4 times the level of antibodies as the asymptomatic surviving mice that received saline (Figure 2C). All mice that lost and regained weight and survived to sacrifice on day 14 developed substantial anti-viral antibodies, at least 10 fold more than the asymptomatic, poorly infected mice (Figure 2D). Correlation analysis of maximum % weight loss and antibody titers for each surviving mice (Figure 2E) shows that mice exhibiting larger virus-induced weight loss elicited a larger immune response (Pearson coefficient=0.71; p=0.0145). In a regression model, with treatment as an additive effect, estimated serum antibody was greater in GM-CSF than in saline by an estimated 1.0969 units on the natural logarithmic scale, or a ratio of 2.99 (p value = 0.2458). Treatment as an effect modifier was considered, but the estimates for the GM-CSF and saline slopes were similar and there was no evidence of a difference between the slopes. The estimated common slope was 1.0144 units on the natural logarithm of serum antibody, or a change of 175.776% in the serum antibody, per 10 percent units of maximum weight loss (p value = 0.0239).

## GM-CSF Reduces Virus-induced Mortality

**Figure 3.**
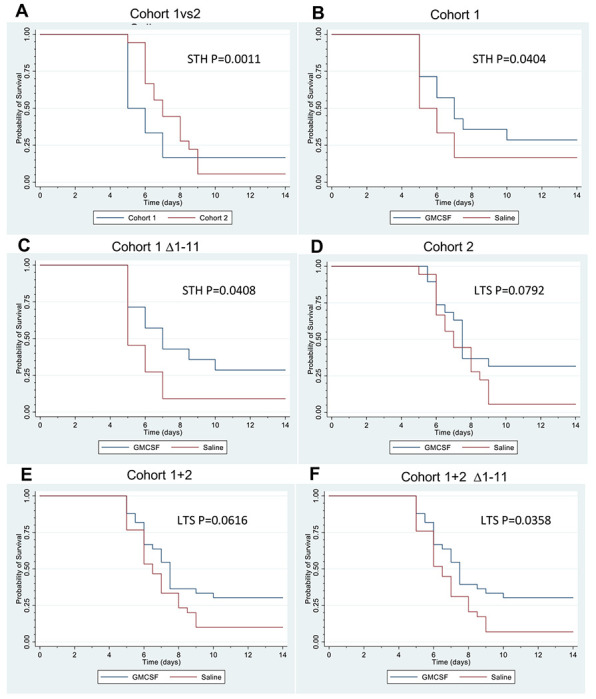
GM-CSF Treatment Significantly Improves Survival in SARS-CoV-2-Infected K18-hACE2 Mice. Mortality data from Tables S1 and S2 are presented as Kaplan-Meier plots. Analyses were carried out with the cure mixture model using a Weibull distribution for the time to event for the non-survivors, censoring mice #1-15 and #2-21 that died immediately after the injection, and excluding mice #1-12, #1-13, #2-19, #2-20, #2-35, and #2-42 that were not effectively infected. **A.** Comparison of mortality in the saline-treated mice in cohorts 1 and 2. The short-term hazard (STH in figure) for saline in cohort 1 was greater than that for cohort 2, by an estimated 459.18% (hazard ratio estimate = 5.5918, 95% CI: (2.1120, 14.7479), p value = 0.0011). The cohort (instillation volume) effect on the long-term survival was statistically non-significant (cohort 1 vs 2: odds ratio estimate for long-term survival = 3.4001, 95% CI: (0.2450, 47.1861), p value = 0.3496). The joint test for a treatment effect on either short-term or long-term survival was statistically significant (p value = 0.0032). **B.** Comparison of mortality in GM-CSF- and saline-treated mice in cohort 1, including the borderline infected mouse #1-11. GM-CSF decreased the short-term hazard, compared to saline, by an estimated 67.20% (hazard ratio estimate = 0.3280, 95% CI: (0.1134, 0.9489), p value = 0.0404). The treatment effect for the long-term survival was statistically non-significant (odds ratio estimate = 1.9999, 95% CI: (0.2697, 14.8292), p value = 0.4833). The joint (long- or short-term) treatment effect test was marginally statistically non-significant (p value = 0.0951). **C.** Leaving out the borderline infected mouse #1-11, GM-CSF treatment decreased the short-term hazard, compared to saline, by an estimated 67.20% (hazard ratio estimate = 0.3280, 95% CI: (0.1132, 0.9509), p value = 0.0408). The joint treatment effect test was marginally statistically non-significant (p value = 0.0686). **D.** Comparison of GM-CSF- and saline-treated mice in cohort 2, in which lung infection was much less than in cohort 1. GM-CSF treatment marginally statistically non-significantly increased the odds of long-term survival (LTS in figure), compared to saline, by an estimated 684.60% (odds ratio estimate = 7.8460, 95% CI: (0.7770, 79.2336), p value = 0.0792). **E.** Comparison of mortality in GM-CSF- and saline-treated mice, combining cohort 1 (including mouse #1-11) and cohort 2. GM-CSF treatment marginally statistically non-significantly increased the odds of long-term survival, compared to saline, by an estimated 291.30% (odds ratio estimate = 3.9130, 95% CI: (0.9341, 16.3921), p value = 0.0616). **F.** Comparison of mortality in GM-CSF- and saline-treated mice, combining cohort 1 (excluding mouse #1-11) and cohort 2. Treatment with GM-CSF increased the odds of long-term survival, compared to saline, by an estimated 486.97% (odds ratio estimate = 5.8697, 95% CI: (1.1284, 30.5328), p value = 0.0358).

Kaplan-Meier survival curves of the mice from the GM-CSF- and saline-treated groups for each cohort separately and combined are shown in Figure 3. Figure 3A shows that saline mice in cohort 1 exhibited >five-fold increased hazard of short-term death (p=0.0011) compared to mice in cohort 2. The joint test for either short- or long-term survival also showed a statistically significant effect (p=0.0032), reflecting the different lung and brain viral titers in mice in the two cohorts. Figure 3B plots mortality of mice in cohort 1, except for mouse #1-15 that died immediately after treatment injection and was censored, and mice #1-12, #1-13, and #1-21, that were poorly infected, as discussed above. GM-CSF treatment reduced the short-term hazard of death by 67.2% (p=0.0404) compared to saline. The joint test for a treatment effect on either short- or long-term survival was marginally statistically non-significant (p=0.0951). Figure 3C shows the Kaplan-Meier plot of the same cohort 1 mice as in B, but excluding mouse #1-11, which was borderline infected, resulting in a small effect on the statistics (p=0.0408 for short term survival and p=0.0686 for the joint test). 3D shows the survival in cohort 2, excluding censored mouse #2-21 and mice #2-19, #2-20, #2-35, and #2-42, which were asymptomatic/poorly-infected based on their lack of both weight loss and few anti-viral antibodies. GM-CSF marginally statistically non-significantly increased the odds of long-term survival, compared to saline, by a very large estimated 684.60% (p=0.0792).

The Kaplan-Meier plots of the combined survival data from the two cohorts are shown in Figure 3E (including mouse #1-11) and Figure 3F (excluding mouse #1-11). When mouse # 1-11 is included in the combined cohorts, GM-CSF treatment increased the odds of long-term survival by an estimated 291.30% compared to saline, with the effect being large and marginally statistically non-significant (p=0.0616). For the combined cohorts, excluding mouse #1-11, GM-CSF treatment statistically significantly increased the odds of long term-survival by an estimated 486.97% compared to saline (p=0.0358). Estimates of the odds ratios for long term survival are imprecise (wide confidence intervals) because of the small number of survivors, specifically only one infected survivor in each of the saline-treated groups.

## Discussion

Compared to saline, daily injections of GM-CSF initiated one day after SARS-CoV-2 infection in susceptible K18-hACE2 mice increased anti-viral antibodies in the sera, reduced viral titers in the lungs, improved long-term survival, and reduced the short-term hazard of death in mice highly infected in the lungs.

GM-CSF/sargramostim/Leukine® has been approved by the FDA for over 30 years to mobilize HSCs and increase white blood cell numbers for various indications^30^. The results presented here validate our hypothesis based on preliminary experiments with WNV-infected mice that rhGM-CSF/sargramostim may be an effective treatment for SARS-CoV-2 infection (COVID-19) in human patients if started early enough to activate both the innate and adaptive immune systems to suppress the viral infection^1^. During the course of these experiments, a preprint reported the results of a small open label trial in which inhaled GM-CSF/sargramostim was used to treat hospitalized COVID-19 patients also being treated with hydroxychloroquine or dexamethasone, resulting in increases in blood oxygenation and anti-virus T-cells, but the sample size was too small to observe an effect on duration of hospitalization or on mortality^31^.

GM-CSF has usually been considered to be a pro-inflammatory cytokine because it induces an increase in the expression of other cytokines associated with inflammation such as IL-1, IL-6, IL-2, and TNF-α, but it also increases the expression of anti-inflammatory cytokines such as IL-10 and decreases IL-8, the prominent chemotactic factor for neutrophils^1,10^. The findings reported here suggest that inflammation per se is not necessarily detrimental, but can be recruited to effectively attack infectious diseases by using GM-CSF, a natural immune system stimulant and modulator. The results also indicate that short-term mortality is most prevalent when the initial inoculum reaches the lungs, with implications for public health measures. Current approaches to the treatment of COVID-19 include anti-virus monoclonal antibodies, drugs designed to prevent viral replication, and broad-acting immunosuppressant corticosteroids. GM-CSF/sargramostim treatment has a major potential advantage over these approaches because it activates specific cells of the endogenous immune system, with likely long-term increases in cellular and humoral immune memory required for protection against future infection, whereas prophylactic ant-viral and broad-acting immunosuppressant treatments only offer transient benefits and likely reduce the long-term immune response and its protection. In principle, GM-CSF treatment should be effective against all SARS-CoV-2 variants.

## Methods

### Mice

K18-hACE2 mice were obtained from Jackson Laboratories (Stock No. 034860), housed and treated in the Colorado State University (CSU) Rocky Mountain Regional Biocontainment BSL3 animal facility in a room with HEPA-filtered air and a 12:12 light:dark cycle, fed Teklad Irradiated Diet 2918 (Envigo, Madison WI), and provided with filter-sterilized water *ad libitum*. All procedures were approved by the CSU Institutional Animal Care and Use Committee (IACUC) and performed in accordance with National Institutes of Health guidelines for the care and use of animals in research. Same-sex littermates were housed in the same cage. In the experiment with cohort 1 mice, eight male and eight female mice were treated with GM-CSF, and seven male and seven female mice were treated with the saline control daily starting at one day post-infection with SARS-CoV-2. In the experiment with cohort 2 mice, 11 male and 11 female mice were treated with GM-CSF, and 10 male and 10 female mice were treated with the saline control daily starting at one day post-infection with SARS-CoV-2. Because some mice in cohort 1 failed to lose weight and survived to the end of the experiment on day 14, the genotypes of all of the cohort 1 mice were double checked by polymerase chain reaction (PCR) using a protocol from the Jackson Laboratory, and all were found to harbor the hACE2 transgene as expected. Thus, it was concluded that mice (#1-12, #1-13, #1-26) that were asymptomatic, did not lose substantial weight, and then survived to the end of the experiment on day 14 were poorly infected, and they were not included in the survival analyses. Based on maximum weight loss of 13.12%, one mouse (#1-11) was considered to have been borderline infected. All other mice that survived in cohort 1 or 2 suffered a maximum weight loss between 15.58%-34.18%. Therefore, survival analyses were calculated separately in which mouse #1-11 was included or excluded. Despite being poorly infected, four surviving mice in cohort 2 #2-19 (saline), #2-20 (saline) #2-35 (GM-CSF), and #2-42 (GM-CSF) did make some (albeit much less) anti-SARS-CoV-2 spike protein antibodies compared to the well-infected mice.

### Virus

SARS-CoV-2 virus strain WA1/2020WY96 was obtained from BEI Resources (Manassas, VA, USA), passaged twice in Vero E6 cells, and stocks were frozen at −80°C in Dulbecco’s Modified Eagle Medium (DMEM) with 5% fetal bovine serum and antibiotics. The virus stock was titrated on Vero cells using a standard double overlay plaque assay^32^, and plaques were counted 72 h later to determine plaque-forming units (PFU) per ml.

### Viral Assays

Live virus particles were detected in homogenized tissue samples by double overlay plaque assay on Vero cells as described previously^33^. Briefly, six-well plates with confluent monolayers of cells were inoculated with 100 μl of 10-fold serial dilutions of samples, incubated for 1 h at 37°C, and overlaid with a 0.5% agarose in MEM containing 2% fetal bovine serum and antibiotics/antifungal agents. A second overlay with neutral red dye was added at 24 h and plaques were counted at 48 h. Viral titers are reported as the PFU per 100 μg lung or brain tissue.

### Antibody Assays

Blood was not collected from cohort 1 mice and was collected from as many mice of cohort 2 as possible (ie. not from mice that were found dead in their cage in the morning or two that were hard to bleed) by cardiac puncture at euthanasia or sacrifice and sera samples were prepared and frozen. Before shipping to the University of Colorado lab, the virus in the sera was inactivated by heat treatment. The antibody levels were determined by a commercial ELISA assay designed to measure mouse antibodies against the SARS-CoV2 spike protein, according to the manufacture’s protocol (KRISHGEN Biosystems Cat No. KBVH015-14). Several dilutions of sera were measured, 1:1000 (recommended), 1:100, and 1:50, and the 450nM absorbance of each sample determined in duplicate, using a SYNERGY H4 plate reader. Company-provided antibody controls were used for each plate to generate standard curves in GraphPad Prism 9 from which the levels of antibody in each serum sample were determined by interpolation.

### GM-CSF

GM-CSF is a glycosylated polypeptide signaling molecule that is typically secreted by immune cells such as macrophages, T cells, mast cells, natural killer (NK) cells, and other tissue cells, such as endothelial cells and fibroblasts. In the bone marrow, GM-CSF functions as a leukocyte growth factor and stimulates hematopoietic progenitor cell mobilization, proliferation, and differentiation towards monocytes, granulocytes, and dendritic cells, as well as inducing endothelial progenitor cells and other myeloid-lineage cells. In the brain, GM-CSF increases the number and activation of microglia, and in addition to its growth factor function, GM-CSF acts as an important modulator of immune responses ^8,9^. Recombinant mouse GM-CSF was obtained from R&D Systems (Minneapolis, MN)

### Procedure

K18-hACE2 mice lose substantial weight and die or require euthanasia by 5-8 days after intra-nasal inoculation with 10^5^ plaque-forming units (PFU) of SARS-CoV-2 virus^22–26^ Some K18-hACE2 animals inoculated with a smaller inoculum (e.g., 2x10^3^ PFU) can survive, despite showing significant weight loss^27^. Experiments used two cohorts of hACE2 transgenic mice. For cohort 1, on day 0, 8-week-old mice were inoculated by nasal application of 10^4^ plaque forming units (PFU) of SARS-CoV2 virus in 50μL of PBS under light anesthesia (intraperitoneal ketamine 100 mg/kg and xylazine 5 mg/kg). Starting 24 hours later (Day1), a treatment regimen consisting of daily intraperitoneal (IP) injections of GM-CSF (200 mcg/kg in 200 μL) or daily IP injection with an equal volume of sterile saline (200 μL) was started in 16 and 14 randomly-chosen mice, respectively. Treatments continued daily, with the last injection being on day 14. Mice were examined twice a day, with their weights recorded in the morning. Mouse survival was scored over the course of the study. Moribund mice that had mouse grimace scores of 2 (Langford et al)^34^ and had lost greater than 20% of their body weight (compared to their weight on the day they were injected with SARS-CoV-2 were euthanized by Intraperitoneal pentobarbital and scored as “non-surviving.” Because some mice were found dead in their cages in the morning (FD), their mortality was assigned to the previous half-day (i.e., night). Mice that remained alive for 14 days (survivors) were sacrificed. At death/euthanasia or sacrifice, brain and lung tissues were collected frozen at −80°F or fixed in fresh 10% buffered formalin.

The data from the first experiment (cohort 1) were analyzed and were found to show that GM-CSF treatment led to a statistically significant slowing of the time of death of the non-survivors, a trend toward increased long-term survival in the survivors that was not statistically significant due to small numbers, and a trend toward reduced lung viral titers at death of the non-survivors. Based on these results, a second cohort experiment was initiated. The only difference between the procedure for cohort 1 and cohort 2 was that in cohort 1, the 10^4^ PFU inoculum was administered in a 50 μL volume, and, because nasal instillation of 50 μL was time-consuming and was thought to perhaps not result in complete instillation, in cohort 2, the same virus inoculum of 10^4^ PFU was instead administered in a 25 μL volume. As is evident in the results, this seemingly minor difference in the administration of the virus led to large differences in the virus titer and mortality results that imply that the severity of COVID-19 in humans is also likely to be greatly influenced by the level of lung infection.

### Statistical Analyses

Most analysis were performed by cohort because of different viral loads. The relatively small sample size restricted the complexity of models which could be fit. Univariate alpha was set to 0.05. p values in the range of (0.05, 0.10) were treated as marginally statistically non-significant because of the small sample size. Multiple testing adjustments were not considered because of the power limitations. Statistics were performed in SAS 9.4, STATA 15.1, and R 3.6.1.

Percent changes in mouse weight by Day Post-Infection (DPI) relative to baseline was tracked with spaghetti plots, and maximum and average percent change across time were recorded. The post-mortem weights were not included. Weight loss was used to determine if mice were adequately infected. Longitudinal regression models across days were fit for the logarithm of weight.

Brain and lung PFUs were compared between treatment groups by cohort on the non-survivors. Undetectable lung and brain PFU measures (< 10 PFU/100μg) were treated as zeros. Compared to the detectable values the range of undetectability was trivial. T-tests compared the means of the treatment groups. The percent difference between the treatment means, compared to the saline mean, was considered as well, both because of the range of magnitudes, and to make it possible to combine the cohorts. The statistical calculations were performed on the logarithm of the ratio of the treatment means, and cohorts were combined by weighting the log ratios by the cohort sample sizes. They were tested with T-tests constructed using the delta method.

The relationship between serum antibody (logarithmic transform) and maximum percent weight change was analyzed with standard correlation and regression methods. The regression models pooled the residual variance across treatment groups because of the small sample size and lack of scatter for the saline group. Treatment as an effect modifier was considered, however the model was simplified to an additive one when the estimated slopes were similar for both treatments, and there was no evidence of effect modification of slope.

Time to death from infection was compared between treatment groups by cohort and with cohorts pooled on the successfully infected mice. Deaths from causes other than infection were censored. Time was right censored at day 14, which was considered recovery and hence long-term survival. Standard time to event analyses, such as Kaplan-Meier, with log rank and Peto test for treatment group differences, and proportional hazards models were considered. Because recovery was a possibility, a cure mixture model was fit as well: combining a binary model of long-term survival, with a Weibull hazard model for short term mortality.

## Supplementary Material

1
