# Supplementary material for: GM-CSF Promotes Immune Response and Survival in a Mouse Model of COVID-19
Source: Res Sq. 2022 Jan 26:rs.3.rs-1213395. Preprint. [Version 1] doi: 10.21203/rs.3.rs-1213395/v1 (PMC8811947; doi:10.21203/rs.3.rs-1213395/v1)
Supplement: 1 [file NIHPPrs1213395v1-supplement-1.pdf]

ID	S	Treat.	DPI 0	DPI 1	DPI 2	DPI 3	DPI 4	DPI 5	DPI 6	DPI 7	DPI 8	DPI 9	DPI 10	DPI 11	DPI 12	DPI 13	DPI 14	Max %Wt Loss	Lung PFU	Brain PFU
1	M	Saline	21.1	21.10	21.6	21.7	20.0	18.1										14.22	188000	3.2E+14
2	F	Saline	17.6	16.90	17.2	17.4	16.8	15.9										9.66	500000	3.4E+14
3	F	Saline	17.2	16.20	16.6	17.1	15.8	13.7										20.35	292000	2.44E+14
4	M	Saline	22.7	22.70	22.4	22.3	20.9	19.4										14.54	500000	2.8E+14
5	F	Saline	17.9	17.80	17.6	17.8	18.3	15.8										11.73	26000	2.6E+14
6	F	Saline	16.1	15.80	16.3	16.1	15.8	14.6										9.32	220000	4E+14
7	F	Saline	15.7	15.30	15.1	15.2	13.9	12.8	11.80									24.84	89000	7.8E+12
8	M	Saline	19.9	18.70	19.2	19.4	17.8	16.9	15.70									21.11	110000	9.2E+11
9	M	Saline	20.1	19.30	21.2	20.3	19.1	17.0	16.20	15.5								22.89	42000	4E+14
10	F	Saline	17.2	17.20	17.1	17.1	17.3	15.6	13.70	14.1								20.35	80000	9.4E+11
11	M	Saline	22.1	21.70	22.5	22.4	21.3	20.3	19.20	19.6	20.7	22.0	22.0	22.1	22.0	22.3	22.7	13.12	0	0
12	M	Saline	23.9	22.90	23.4	24.9	24.8	24.8	25.30	25.3	25.3	25.9	25.6	25.8	25.7	25.9	25.9	4.18		
13	M	Saline	21.7	21.36	21.2	21.4	21.5	21.2	20.00	19.8	20.4	21.5	21.4	21.4	21.4	21.5	21.7	8.76	0	0
14	F	Saline	16.9	16.60	16.6	16.5	16.5	16.4	14.90	14.3	15.4	15.9	16.7	16.9	17.2	17.6	17.3	15.38	0	0
15	F	GMCSF	19.0	18.30	18.3	*17.8												6.32	500000	9E+12
16	M	GMCSF	21.3	21.20	21.3	21.6	19.4	19.6										8.92	78000	1.5E+14
17	M	GMCSF	20.8	19.70	19.8	19.1	17.2	16.5										20.67	180000	2.42E+14
18	F	GMCSF	15.8	15.70	15.6	15.8	15.4	13.6										13.92	54000	2.4E+14
19	F	GMCSF	17.4	17.00	17.1	16.9	14.7	13.5										22.41	140000	3.64E+14
20	M	GMCSF	20.2	20.70	21.0	21.2	18.6	17.6	16.60									17.82	44000	1.48E+14
21	F	GMCSF	14.9	14.80	15.5	15.4	15.2	14.3	14.20									4.70	146000	7.9E+13
22	M	GMCSF	18.4	17.80	18.3	18.4	18.1	17.4	16.40	15.4								16.30	16000	3.6E+14
23	F	GMCSF	15.8	15.60	15.7	15.7	15.4	13.6	12.90	12.2								22.78	67000	2.4E+14
24	M	GMCSF	20.9	20.20	20.5	21.1	21.1	19.6	18.10	16.8	16.0	FD						23.45	0	130
25	F	GMCSF	17.6	17.80	17.8	17.5	16.2	15.8	14.50	13.8	13.1	13.2	12.5					28.98	0	43000
26	M	GMCSF	22.1	22.30	22.3	22.3	23.0	22.6	22.40	22.6	22.2	22.6	22.3	22.5	22.7	22.3	23.4	-0.45		
27	M	GMCSF	21.4	21.60	21.4	21.5	21.5	20.4	18.36	17.7	19.0	20.8	21.3	21.8	22.3	22.8	23.9	17.29	0	0
28	M	GMCSF	22.1	21.70	21.7	21.9	20.2	19.6	18.50	19.5	20.7	21.9	22.3	22.3	22.4	22.9	23.2	16.29	0	0
29	F	GMCSF	19.2	19.40	19.2	19.0	18.5	16.4	15.50	14.1	14.6	16.2	17.5	17.6	17.9	18.4	18.6	26.56	0	0
30	F	GMCSF	18.2	17.80	18.0	18.5	17.7	16.8	15.50	14.6	14.8	16.2	17.0	17.9	18.3	18.8	18.7	19.78	0	0

Table S1. Cohort 1 mice weights (g) at each day living post infection (DPI) and viral titers(PFU/100 μg of lung or brain tissue) at death, euthanasia, or sacrifice at 14d. Daily GM-CSF treatment began one day after infection. FD: Found Dead; death ascribed to the previous night. *indicates a mouse that died from the injection itself and is censored in subsequent analyses.

ID	S	Treat.	DPI 0	DPI 1	DPI 2	DPI 3	DPI 4	DPI 5	DPI 6	DPI 7	DPI 8	DPI 9	DPI 10	DPI 11	DPI 12	DPI 13	DPI 14	Max. %Wt Loss	Lung PFU	Brain PFU	Serum Antibody ng/100µL
1	M	Saline	24.3	24.0	24.5	24.6	24.2	22.6										7.00	0	44000000	ND
2	F	Saline	18.9	18.6	18.9	18.6	18.0	16.3	15.4									13.76	4000	4300000	ND
3	F	Saline	16.5	16.6	16.3	16.4	15.5	14.1	13.4									18.79	1500	79000000	21
4	F	Saline	17.8	17.4	17.8	17.2	15.8	14.7	14.3									19.66	25000	51000000	66
5	M	Saline	20.5	20.2	19.8	19.4	17.6	16.4	15.8									22.93	46000	90000000	6
6	M	Saline	20.9	20.5	20.5	20.8	21.3	20.4	20.0									4.31	2E+05	71000000	ND
7	F	Saline	18.3	17.9	17.3	17.4	16.3	15.0	14.4	13.8								21.31	22000	84000000	NA
8	M	Saline	21.5	21.0	20.6	20.6	20.0	19.5	17.8	FD								17.21	0	0	NA
9	F	Saline	18.2	17.9	18.0	17.9	16.1	14.9	15.6	12.8								29.67	0	95000000	43
10	M	Saline	26.1	25.6	25.0	25.0	25.4	24.1	23.2	15.2								41.76	17000	3000000	ND
11	F	Saline	18.4	18.0	18.5	17.8	16.7	15.6	14.8	14.2	13.7							25.54	0	0	107
12	F	Saline	17.8	17.8	18.2	18.0	17.4	15.9	14.7	14.3	13.5							24.16	0	0	ND
13	F	Saline	19.3	19.3	18.7	18.5	17.7	16.5	15.4	14.5	14.1							26.94	0	46000000	NA
14	M	Saline	23.7	23.4	23.0	23.0	23.2	22.5	20.2	19.4	18.4	FD						22.36	0	0	NA
15	F	Saline	18.7	18.1	18.2	18.5	18.6	17.3	16.2	18.4	14.8							20.86	0	0	466
16	M	Saline	21.6	21.3	21.0	21.2	19.9	18.4	17.2	16.7	16.0	16.1						25.93	0	0	199
17	M	Saline	24.5	24.2	24.0	23.9	22.1	20.8	19.8	18.8	18.4	17.9						26.94	0	0	ND
18	F	Saline	18.8	18.2	17.8	17.5	15.6	14.6	13.9	13.2	13.4	13.3	14.5	15.1	15.9	16.8	17.8	29.79	0	0	578
19	M	Saline	24.3	23.7	23.9	24.4	24.3	23.9	24.3	24.3	24.6	24.5	24.3	24.3	24.6	25.1	26.4	2.47	0	0	32
20	M	Saline	24.4	24.3	24.1	24.1	24.3	23.7	24.8	24.6	24.8	25.0	25.2	25.1	25.1	25.4	26.2	2.87	0	0	38
21	F	GM-CSF	16.5	16.1	16.5	16.5	15.9	*14.5										12.12	27000	1.11E+08	ND
22	F	GM-CSF	18.3	17.5	17.7	17.7	16.2	14.8	FD									19.13	14000	1.07E+08	NA
23	M	GM-CSF	23.3	23.5	23.2	22.9	20.6	18.7	FD									19.74	1200	70000000	NA
24	M	GM-CSF	22.9	23.1	22.8	23.2	20.3	18.8	17.8									22.27	0	4100000	ND
25	M	GM-CSF	24.4	23.8	23.6	23.8	21.3	20.0	19.8									18.85	2200	1.1E+08	ND
26	M	GM-CSF	22.1	21.7	21.8	22.1	22.2	20.5	19.7									10.86	2000	52000000	ND
27	M	GM-CSF	18.0	17.5	17.7	17.4	15.8	14.7	14.1	FD								21.67	200	0	NA
28	M	GM-CSF	21.8	21.3	21.5	21.0	19.3	17.9	16.9	16.0								26.61	4000	79000000	ND
29	F	GM-CSF	17.3	16.8	17.1	16.6	15.1	14.1	13.3	12.7	FD							26.59	0	0	NA
30	F	GM-CSF	18.5	18.7	18.2	18.6	17.3	15.9	15.2	14.5	FD							21.62	0	0	NA
31	F	GM-CSF	18.4	18.2	17.6	17.6	16.3	15.2	14.6	14.3	FD							22.28	0	0	NA
32	M	GM-CSF	20.0	19.6	19.8	20.1	19.5	18.0	17.1	16.1	FD							19.50	0		NA
33	M	GM-CSF	22.7	22.1	22.2	22.1	20.0	19.2	17.8	17.2	FD							24.23	0	0	NA
34	F	GM-CSF	18.0	17.6	17.6	17.3	16.8	15.5	14.7	13.8	13.4	12.6						30.00	0	0	76
35	F	GM-CSF	17.6	17.1	17.4	17.5	17.2	17.5	17.6	17.8	18.0	18.1	18.2	18.5	18.5	18.4	18.8	2.84	0	0	64

36	F	GM-CSF	16.8	16.7	17.2	17.3	17.4	16.5	15.2	14.4	13.6	14.5	15.6	16.3	16.8	17.8	18.2	19.05	0	0	758
37	F	GM-CSF	19.9	19.5	19.9	19.9	19.8	19.3	17.6	16.7	17.1	17.7	18.4	18.6	18.6	18.9	19.6	16.08	0	0	624
38	F	GM-CSF	18.7	18.5	18.2	18.4	18.4	17.0	15.7	14.8	15.5	16.7	17.0	18.2	18.7	18.7	18.8	20.86	0	0	1178
39	F	GM-CSF	17.6	17.4	17.2	17.3	17.3	16.4	15.1	14.1	13.8	14.8	16.0	16.5	16.5	16.9	17.6	21.59	0	0	7879
40	M	GM-CSF	23.1	23.0	22.8	22.9	22.8	22.2	20.6	19.5	19.6	20.9	21.7	22.4	22.7	23.2	24.2	15.58	0	0	522
41	M	GM-CSF	23.7	22.8	22.5	21.9	19.7	18.7	17.8	17.4	16.9	15.6	16.4	16.2	16.6	16.6	18.8	34.18	0	0	297
42	M	GM-CSF	23.4		22.5	22.6	22.8	23.2	23.0	23.2	23.6	23.7	23.8	23.2	23.3	23.1	24.6	3.85	0	0	36

Table S2. Cohort 2 mice showing weights (g) at each day that they are alive post infection (DPI), viral titers (PFU/100 μg of lung or brain tissue), and anti-CoV-2 spike protein antibodies in sera (ng/mL) at death, euthanasia or sacrifice at 14d. Daily GM-CSF treatment began one day after infection. NA:Blood not collectable. FD: Found Dead; death ascribed to the previous night. *indicates a mouse that died from the injection itself and is censored in subsequent analyses.
